# Dr. Felix E. Brown

**Published:** 1896-06

**Authors:** 


### DR. FELIX E. BROWN.

Dr. Felix E. Brown died Thursday, April 16th, at his home, Preston street near Aisquith, from Bright's disease, after a short illness.

He was born in Baltimore fifty-two years ago, and was a son of the late Dr. A. J. Brown, who graduated at the Baltimore College of Dental Surgery in 1853. The son followed his father's profession.

During the civil war he served in the Union army. He was a member of Post 46, Grand Army of the Republic, and also a member of the Ancient Order of United Workmen. A wife and four children survive him.

